# Optimization of the examination posture in spinal curvature assessment

**DOI:** 10.1186/1748-7161-7-10

**Published:** 2012-04-30

**Authors:** Jakub Krejci, Jiri Gallo, Petr Stepanik, Jiri Salinger

**Affiliations:** 1 Department of Natural Sciences in Kinanthropology, Faculty of Physical Culture, Palacky University, Olomouc, Czech Republic; 2 Department of Orthopedics, University Hospital, Faculty of Medicine and Dentistry, Palacky University, Olomouc, Czech Republic

**Keywords:** Spinal deformity, Examination posture, Postural sway, Non-invasive assessment, DTP-3 system, Approximation polynomial

## Abstract

To decrease the influence of postural sway during spinal measurements, an instrumented fixation posture (called G) was proposed and tested in comparison with the free standing posture (A) using the DTP-3 system in a group of 70 healthy volunteers. The measurement was performed 5 times on each subject and each position was tested by a newly developed device for non-invasive spinal measurements called DTP-3 system. Changes in postural stability of the spinous processes for each subject/the whole group were evaluated by employing standard statistical tools. Posture G, when compared to posture A, reduced postural sway significantly in all spinous processes from C3 to L5 in both the mediolateral and anterioposterior directions. Posture G also significantly reduced postural sway in the vertical direction in 18 out of 22 spinous processes. Importantly, posture G did not significantly influence the spinal curvature.

## Background

Assessment of spinal deformity, especially adolescent idiopathic scoliosis, is generally performed radiologically along with evaluation of the spinal curvature using the Cobb method. Although radiography is the golden standard in orthopaedic practice, it carries health risk from exposure to ionizing radiation [1]. To this end, radiography seems to be unsuitable for screening spinal deformity in its early stages and moreover, it is risky when used for repeated monitoring after conservative or surgical therapy [2]. Therefore, various examination methods enabling non-invasive spinal curvature assessment have been developed [3-7]. However, these methods have not gained widespread use in clinical practice as yet. The main problem may be in the relatively low correlation between radiographic and non-invasive spinal curvature measurements [8].

It is well-known that maintaining a standing posture can be compromised by postural sway, which manifests as random deflections of each body segment, especially in children and adolescents [9,10]. From the perspective of reliability of spinal shape examination, postural sway is an undesirable phenomenon since the spinal shape is not depicted in time by a constant curve, but rather by a curve that is to a certain extent continuously changing. It is then impossible to achieve reliable consistency in the results at repeated spinal shape examinations using either radiographic or non-invasive methods. Therefore, postural sway can be one of the explanations for random errors, reducing the reliability of the spinal shape examination [9,11,12]. From the perspective of random errors at examination, it is desirable to reduce postural sway by as much as possible. The size of postural sway depends on the individual control processes of movement, as well as on the particular posture that the subject undergoing examination is adopting (free standing, standing with additional mechanical fixation, sitting, lying, etc.), [13,14]. However, any fixation procedure that reduces the size of postural sway may change the body position and the spinal shape simultaneously. It is obvious that in different examination postures the spinal shape may vary [15,16]. From the perspective of systematic errors at examination, it is important to deal with the question of the extent to which the spinal shape in examination postures with mechanical fixation resembles the spinal shape in free standing posture. Therefore, it is important to determine the characteristics of selected examination posture in terms of both the postural sway and the influence on spinal shape. Theoretically, precise spinal shape examination requires an examination posture that minimizes postural sway (random errors) and the changes in spinal shape (systematic errors). In our previous studies, we revealed that none of the tested postures complies entirely with the above-mentioned prerequisites [15,17]. Appropriate examination posture and standardization of positioning the subject in the course of examination enable mutual comparison of the results of examinations when performed using different methods, e.g. radiographic and non-invasive examinations.

In this study, a new measurement standing posture with an additional fixation frame is described and compared to the free standing posture. The aim is to evaluate the stability of spinous processes during measurement by the DTP-3 system [18] and to determine the influence of the new examination posture on the spinal shape.

## Methods

### Description of fixation frame and fixation posture

**Figure 1:**
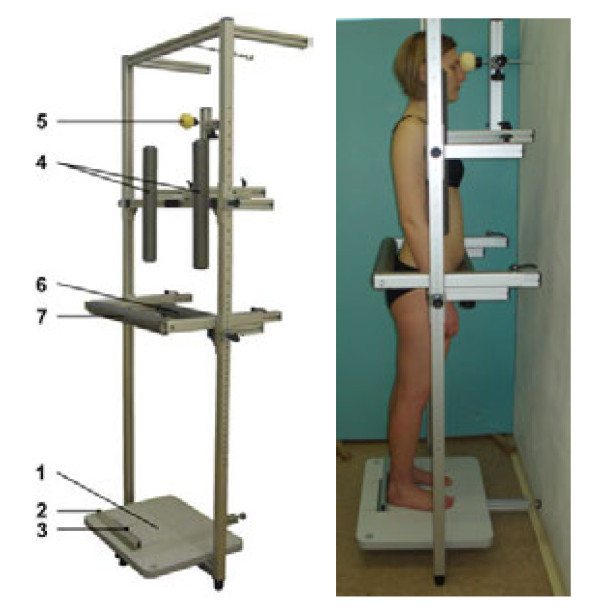
**Fixation frame and fixation posture G.** 1 – stepping platform 2 – ruler for determining the beginning of the ideal vertical 3 – origin of the ideal vertical 4 – shoulder rests 5 – head rest 6 – front pelvis rest 7 – rear pelvis rest.

Fixation posture G is derived from free standing posture, which is supplemented with further fixation in order to reduce postural sway and thus increase reliability of the spinal shape examination. A prototype of the fixation frame was designed and constructed for fixing the subject in the course of the spinal shape examination. The fixation frame (Figure 1) consists of a stepping platform, a support construction and five adjustable rests. Two rests support the front parts of the shoulders and are adjustable in all three directions (vertical, mediolateral and anterioposterior). One of the rests supports the root of the nose and is also adjustable in all three directions. The remaining two rests support the pelvis, fixing the front and rear parts respectively, and are adjustable in the vertical and anterioposterior directions. In order to construct a prototype of the fixation frame, we applied the MayTec modular elements (MayTec, Dachau, Germany). Construction works, based on our requirements, were carried out by the company Amtek (Brno, Czech Republic). The fixation frame can be dismantled by means of couplings and in its longest part it is 1.1 m in length, which enables easy transport in the boot of a car in the event of field examination.

Positioning the subject in the fixation frame is carried out as follows: the subject steps into the fixation frame and assumes the free standing posture. A ruler for determining the beginning of the ideal vertical is placed so that it touches the calcanei and the zero mark of the ruler is placed in the centre of a connecting line between the calcanei. The height and width of the shoulder rests are then adjusted by sliding out the rests so that there is 30 mm between the rests and the front part of the shoulders in the anterioposterior direction. The subject is asked to bend forward and lean his/her shoulders against the rests and not to change the posture any more. The head rest is brought into play so that it gently touches the root of the nose without changing the head position. The front rest of the pelvis gets pushed towards the front part of the pelvis in the area of the anterior superior iliac spines and, finally, the rear pelvis rest is pushed towards the rear part of the pelvis just below the posterior superior iliac spines.

### The instrument

**Figure 2:**
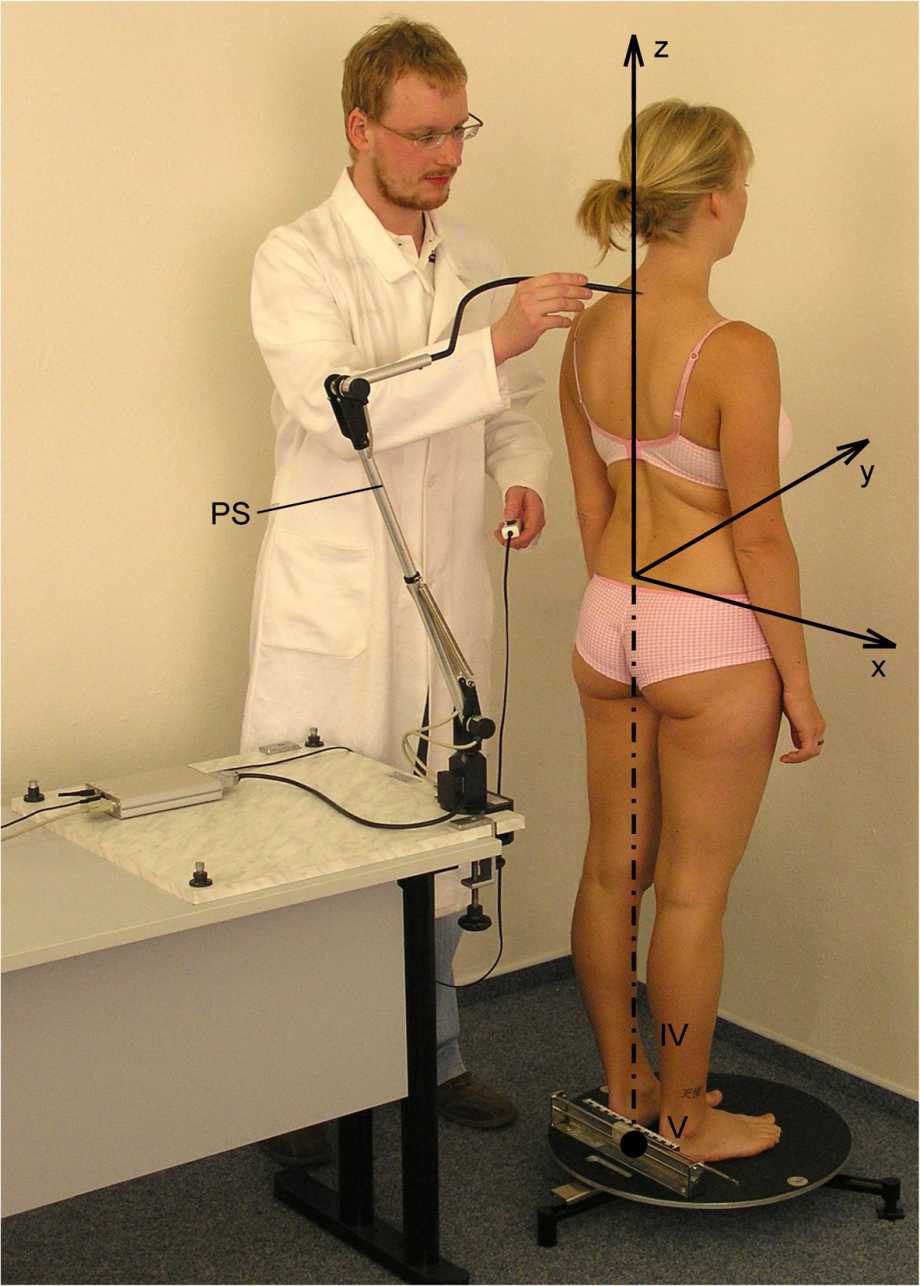
**Examination of spinal shape in the free standing posture using the DTP-3 system.** PS – DTP-3 position sensor V – centre of the intercalcaneal line – origin of the ideal vertical IV – ideal vertical x, y, z – coordinate axes.

The DTP-3 system (Palacky University, Olomouc, Czech Republic) (Figure 2) was developed primarily for non-invasive contact-type assessment of spinal deformity in the sagittal and frontal planes. The measurement is based on determining the three-dimensional (3D) coordinates of points on the skin surface by means of an electromechanical position sensor. Data is transmitted to a computer for subsequent processing. The position sensor consists of a mechanical pantograph with three incremental encoders. The measuring stylus of the position sensor ends in a hemisphere of radius of 1 mm. The position sensor allows measurement of the points with standard error of 0.5 mm in the sphere of 2,200 mm diameter [18].

The so-called ideal vertical (IV), i.e. mathematical simulation of a plumb line erected from the centre of the connecting line between the calcanei, is used to evaluate the spinal balance. The orientation of the 3D Cartesian coordinate system is as follows: axis *z* is on the ideal vertical and oriented in the caudal–cranial direction, axis *x* is parallel to the inter-calcaneal line and in the left-right direction, and axis *y* is in the posterior–anterior direction. As a result, the frontal plane is defined by axes *xz* and the sagittal plane by axes *yz*.

Before using the DTP-3 system for examining spinal shape, the skin projections of the following anatomic points are palpated and marked: the lateral parts of the acromions, the posterior superior iliac spines (PSIS), and the spinous processes of the vertebrae C3–C7, T1–T12, and L1–L5. After positioning the subject, the marked points are scanned by touching them with the position sensor stylus.

### The software for spinal shape evaluation

Direct assessment of 3D coordinates *x*_*i*_*y*_*i*_*z*_*i*_ of all spinous processes, *i* = 1, 2, …, 22, for each subject is time-consuming. Therefore, special DTP-3 software was used to evaluate the spinal shape. The heart of the algorithm is fitting the six degrees polynomial to the measured points. A new normalized coordinate *Z* which is the coordinate *z* scaled to the interval [−1, 1] is introduced. The lowest spinous process L5 has the height of −1 and the highest spinous process C3 has the height of 1. The formula of the six-degree polynomial in the sagittal plane is

(1) y=b0+b1Z+b2P2(Z)+b3P3(Z)+b4P4(Z)+b5P5(Z)+b6P6(Z),

where $\text{P}2(Z)=\frac{3}{2}Z^{2}-\frac{1}{2}$$\text{P}3(Z)=\frac{5}{2}Z^{3}-\frac{3}{2}Z^{}$$\text{P}4(Z)=\frac{35}{8}Z^{4}-\frac{15}{4}Z^{2}+\frac{3}{8}\text{,}$$\text{P}5(Z)=\frac{63}{8}Z^{5}-\frac{35}{4}Z^{3}+\frac{15}{8}Z$ and $\text{P}6(Z)=\frac{231}{16}Z^{6}-\frac{315}{16}Z^{4}+\frac{105}{16}Z^{2}-\frac{5}{16}\text{,}$ are the Legendre polynomials [19]. This scaling and orthogonal procedure increases the numerical stability and reduces the influence of the rounding-off error. The clinical interpretation of polynomial coefficients in the sagittal plane is as follows:

· *b*_0_ – anterioposterior shift of the spine from IV

· *b*_1_ – anterioposterior tilt of the spine to IV

· *b*_2_ – overall spinal curvature (i.e. primarily the curvature of thoracic kyphosis)

· *b*_3_ and *b*_4_ – curvature of the upper and lower parts of the spine (i.e. the curvature of cervical and lumbar lordosis)

· *b*_5_ and *b*_6_ – residual spinal curvature of just units of millimetres

**Figure 3:**
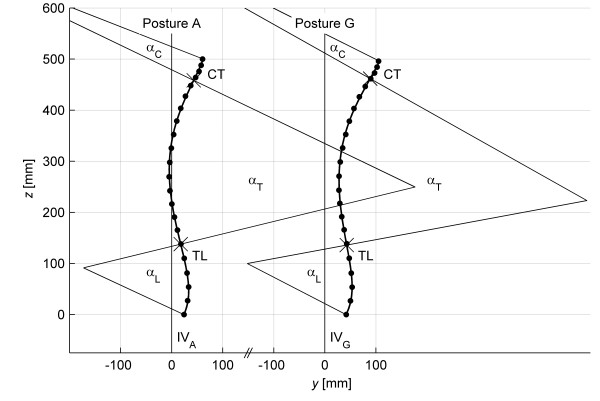
**Mean spinal shapes and means of angle parameters for postures A and G.** Posture A – free standing position Posture G – standing position supported by a fixation frame CT – cervicothoracic junction TL – thoracolumbar junction α_*C*_ – angle of cervical lordosis α_*T*_ – angle of thoracic kyphosis α_*L*_ – angle of lumbar lordosis IV– ideal vertical.

A six-degree polynomial was chosen to describe spinal shape in the sagittal plane since it represented the best approximation to the physiological curvature of the spine with two inflexion points [18]. The positions of the two inflexion points might be interpreted as a cervicothoracic junction (CT) and a thoracolumbar junction (TL). These junctions split the spine into three sections: the cervical, the thoracic and the lumbar spines. The curvature of the respective spinal section could be described with angle parameter (Figure 3) defined as follows:

· αC – the cervical lordosis curvature is the angle between the normal lines (perpendicular line to the tangent of the polynomial curve) projected from the spinous processes at C3 and the CT junction.

· αT – the thoracic kyphosis curvature is the angle between the normal lines projected from the CT junction and the TL junction.

· αL - the lumbar lordosis curvature is the angle between the normal lines projected from the TL junction and the spinous processes L5.

Calculation of the sagittal shift and sagittal tilt is also available and the procedure is as follows: The centre point between the left and right PSIS is calculated. The sagittal shift is the anteroposterior displacement of the centre point from IV. The sagittal tilt is the angle between IV and the connecting line between the centre point and spinous processes C7 [20]. Such calculation produces more straightforward output than the polynomial coefficients *b*_0_ and *b*_1_.

The DTP-3 software that utilizes the abovementioned algorithm was validated by means of x-ray examination. When using a rigid model of the human spine, good concordance between non-invasive DTP-3 and traditional x-ray Cobb angles was demonstrated [21].

### Study group

The experimental part of the study included 70 subjects, 33 men and 37 women, aged 23.4 ± 3.0 years (mean ± SD), weight 70.5 ± 10.4 kg, height 174.2 ± 8.6 cm. The height of the spine given by the vertical distance between the spinous processes of C3 and L5 was 49.4 ± 3.1 cm. The group included healthy students from the Faculty of Physical Culture of Palacky University without any spinal disorders. The study was approved by the Ethical Committee of the Faculty of Physical Culture of Palacky University. All of the subjects participating in this study were volunteers and had given their informed consent.

### Measurement protocol and statistics

Measurement of the marked points was repeated five times for each of the two postures (A – free standing posture, G – standing posture in fixation frame) and individual measurements followed immediately in succession. The duration of one measurement was less than 30 s.

The postural sway of each spinous process was evaluated for each examined subject by way of standard deviations *SD*_*x*_*SD*_*y*_*SD*_*z*_ according to the formulas

(2) $$SDx=\sqrt{\frac{1}{5-1}\sum_{i=1}^{5}{\left(\bar{x}-xi\right)}^{2}}\text{,}\;SDy=\sqrt{\frac{1}{5-1}\sum_{i=1}^{5}{\left(\bar{y}-yi\right)}^{2}}\text{,}\;SDz=\sqrt{\frac{1}{5-1}\sum_{i=1}^{5}{\left(\bar{z}-zi\right)}^{2}}$$

in which *x*_*i*_*y*_*i*_*z*_*i*_ are the coordinates of the spinous process in *i*-th repetition of the measurement (measurement was repeated five times in the selected posture), $\bar{x}\text{,}\;\bar{y}\text{,}\;\bar{z}$ are the mean coordinates of the spinous process. For evaluating postural sway of each spinous process within the group of 70 subjects, examined in a selected posture, means of standard deviations *MSD*_*x*_*MSD*_*y*_*MSD*_*z*_ were calculated as average values of standard deviations *SD*_*x*_*SD*_*y*_*SD*_*z*_ in the entire group [15,17].

Calculations for evaluating postural sway and spinal shape were performed using MATLAB 7.6 (MathWorks, Natick, MA) and STATISTICA Cz 8.0 (StatSoft, Prague, Czech Republic).

## Results

**Figure 4:**
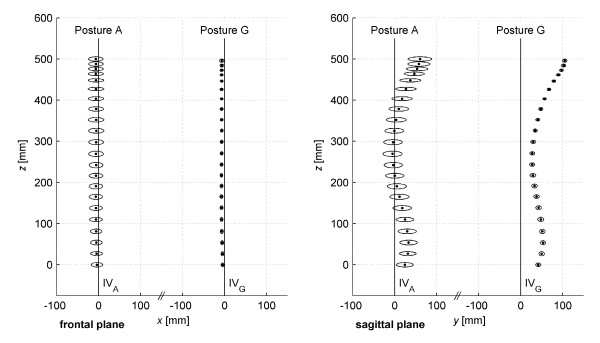
**Mean positions and mean standard deviations of the spinous processes calculated from group of 70 subjects.** Posture A – free standing position Posture G – standing position supported by a fixation frame IV – ideal vertical · – mean position of spinous processes ○ – mean of standard deviations (enlarged five times).

The results of the analysis of postural sway are shown in the Table 1 and Figure 4. Posture G, compared to posture A, reduces (in statistical significance) postural sway in all spinous processes from C3 to L5 in the mediolateral and anterioposterior directions. Reduction in postural sway in the vertical direction is statistically significant for 18 out of 22 spinous processes. The average value of postural sway in posture A is 3.4, 4.6 and 1.1 mm (mediolateral, anterioposterior and vertical direction) respectively. Posture G reduces postural sway to values of 0.8, 1.1 and 0.8 mm respectively.

**Table 1:** Assessment of postural sway of processus spinosus by way of standard deviations calculated from group of 70 subjects

		***x* coordinate**			***y* coordinate**			***z* coordinate**		
**Processus spinosus**		**Posture**		**Comp.**	**Posture**		**Comp.**	**Posture**		**Comp.**
		**A**	**G**	**G – A**	**A**	**G**	**G – A**	**A**	**G**	**G – A**
		**MSD**	**MSD**	**Δ**	**MSD**	**MSD**	**Δ**	**MSD**	**MSD**	**Δ**
C3	[mm]	3.5	1.1	−2.4*	5.7	1.1	−4.6*	1.1	0.8	−0.3*
C4	[mm]	3.5	0.9	−2.5*	5.3	1.1	−4.2*	1.1	0.8	−0.3*
C5	[mm]	3.5	0.8	−2.7*	5.1	1.0	−4.1*	0.9	0.6	−0.3*
C6	[mm]	3.6	0.8	−2.8*	4.8	0.9	−3.9*	0.9	0.6	−0.3*
C7	[mm]	3.7	0.7	−3.0*	5.0	0.9	−4.1*	0.8	0.6	−0.2*
T1	[mm]	3.8	0.8	−3.0*	4.9	0.9	−4.0*	0.9	0.6	−0.3*
T2	[mm]	3.6	0.7	−2.9*	4.8	0.8	−4.0*	1.0	0.6	−0.4*
T3	[mm]	3.6	0.8	−2.8*	4.7	1.0	−3.7*	1.1	0.7	−0.4*
T4	[mm]	3.7	0.7	−3.0*	4.7	1.0	−3.7*	1.1	0.7	−0.4*
T5	[mm]	3.7	0.7	−3.0*	4.5	1.0	−3.5*	1.2	0.8	−0.4*
T6	[mm]	3.6	0.8	−2.8*	4.5	1.2	−3.3*	1.2	0.8	−0.4*
T7	[mm]	3.6	0.7	−2.9*	4.6	1.2	−3.4*	1.3	0.8	−0.5*
T8	[mm]	3.3	0.7	−2.6*	4.4	1.3	−3.1*	1.3	0.9	−0.4*
T9	[mm]	3.3	0.7	−2.6*	4.4	1.3	−3.0*	1.2	0.9	−0.3*
T10	[mm]	3.3	0.7	−2.6*	4.6	1.3	−3.3*	1.3	1.0	−0.3*
T11	[mm]	3.2	0.8	−2.4*	4.6	1.3	−3.3*	1.3	0.9	−0.4*
T12	[mm]	3.1	0.8	−2.3*	4.4	1.3	−3.1*	1.2	1.0	−0.2*
L1	[mm]	3.1	0.7	−2.4*	4.3	1.3	−3.0*	1.1	1.0	−0.1
L2	[mm]	2.8	0.7	−2.1*	4.4	1.3	−3.1*	1.2	1.0	−0.2
L3	[mm]	2.8	0.7	−2.1*	4.2	1.3	−2.9*	1.2	1.0	−0.2
L4	[mm]	2.8	0.7	−2.1*	4.0	1.3	−2.7*	1.1	1.0	−0.1
L5	[mm]	2.7	0.7	−2.0*	4.0	1.2	−2.8*	1.1	0.9	−0.2*
Average		3.4	0.8	−2.6	4.6	1.1	−3.5	1.1	0.8	−0.3

*x* – mediolateral direction.

*y* – posterioanterior direction.

*z* – vertical.

Posture A – free standing position.

Posture G – standing position supported by a fixation frame.

MSD – mean of standard deviations.

Δ – difference of parameters.

* – statistically significant (*p* < 0.05, paired *t*-test).

The results of the analysis of influence of postural sway on spinal measurements are shown in Table 2. In this analysis, each individual measurement of the marked points was fed to the polynomial algorithm and spinal measurements were duly obtained. SDs calculated from five times repeated measurements were used for assessing the influence of postural sway. In this case, SDs represent random error caused by postural sway. Due to the fact that the size of postural sway is subject dependent, MSDs were used for assessing the entire group of 70 subjects. Based on Table 2, posture G compared to posture A reduces MSDs in all of the applied spinal measurements. The values of MSDs of all spinal curvatures were below 1° in posture G.

**Table 2:** Influence of postural sway on spinal measurements

**Parameter**		**Posture**		**Comp.**
		**A**	**G**	**G – A**
		**MSD**	**MSD**	**Δ**
shift	[mm]	2.6	0.8	−1.8*
tilt	[°]	0.3	0.1	−0.2*
α_*C*_	[°]	1.8	0.8	−1.0*
α_*T*_	[°]	1.5	0.5	−0.9*
α_*L*_	[°]	1.4	0.4	−0.9*

Posture A – free standing position.

Posture G – standing position supported by a fixation frame.

MSD – mean of standard deviations.

Δ – difference of parameters.

shift – sagittal shift.

tilt – sagittal tilt.

α_*C*_ – angle of cervical lordosis.

α_*T*_ – angle of thoracic kyphosis.

α_*L*_ – angle of lumbar lordosis.

* – statistically significant (*p* < 0.05, paired *t*-test).

The results of bias analysis of the spinal measurements are shown in Table 3 and Figure 3. In this analysis, the mean coordinates of the marked points were calculated from five times repeated measurements and then the mean coordinates were fed to the polynomial algorithm. The averaging procedure reduces the influence of postural sway on spinal measurements. In this case, SD represents the range of spinal measurement within the group of subjects. Differences in angles α_C_, α_T_ and α_L_ between postures A and G are not statistically significant and the maximum absolute value of the difference is 1.2°. These angle parameters describe, respectively, the curvatures of the cervical, thoracic and lumbar spines. Differences in the sagittal shift and the sagittal tilt between postures A and G are statistically significant. In posture G, the spine (trunk) moved forward in the anteroposterior direction by 15.3 mm and increased flexion by 3.2°. The same results can be obtained after polynomial coefficients analysis. Coefficients *b*_0_ and *b*_1_ that represent the shift and tilt of the spine are statistically different between postures A and G. Coefficients *b*_2_, *b*_3_, *b*_5_ and *b*_6_ that deal with the curvature of the spine are not statistically different. The change of coefficient *b*_4_ is statistically significant but the difference has clinically insignificant value.

**Table 3:** Spinal measurements and polynomial coefficients calculated from group of 70 subjects

**Parameter**		**Posture A**		**Posture G**		**Comparison G − A**	
		**M**	**SD**	**M**	**SD**	**Δ**	***P***
shift	[mm]	16.8	21.7	32.1	18.2	15.3	<0.001*
tilt	[°]	2.4	1.9	5.7	2.1	3.2	<0.001*
α_*C*_	[°]	7.8	8.8	7.1	9.0	−0.7	0.261
α_*T*_	[°]	44.2	8.7	45.4	9.2	1.2	0.070
α_*L*_	[°]	39.3	12.2	38.6	10.3	−0.7	0.305
*b*_0_	[mm]	16.8	19.2	47.6	18.2	30.8	<0.001*
*b*_1_	[mm]	1.9	8.2	15.0	8.3	13.1	<0.001*
*b*_2_	[mm]	36.2	9.7	35.9	9.7	−0.3	0.496
*b*_3_	[mm]	16.8	6.4	17.3	6.6	0.5	0.085
*b*_4_	[mm]	−8.9	3.5	−8.0	3.7	0.9	<0.001*
*b*_5_	[mm]	0.4	3.1	0.6	2.9	0.2	0.197
*b*_6_	[mm]	−1.2	2.2	−1.2	2.3	0.0	0.877

Posture A – free standing position.

Posture G – standing position supported by a fixation frame.

M – arithmetic mean.

SD – standard deviation.

Δ – difference of parameters.

*P* – significance of paired *t*-test.

shift – sagittal shift.

tilt – sagittal tilt.

α_*C*_ – angle of cervical lordosis.

α_*T*_ – angle of thoracic kyphosis.

α_*L*_ – angle of lumbar lordosis.

*b*_0_, *b*_1_,…, *b*_6_ – polynomial coefficients in the sagittal plane.

* – statistically significant (*p* < 0.05).

Taken together, our results can be interpreted as posture G reducing postural sway and subsequently reducing random error in all spinal measurements. Compared to posture A, posture G does not significantly bias the curvatures of the spine but it biases the sagittal shift and the sagittal tilt.

## Discussion

Surprisingly, when looking at the literature there is only little attention devoted to the evaluation of an examination posture with regard to a particular spinal shape measurement method [11,22]. Selecting the most appropriate examination posture does not depend on the examination method, and hence this is applicable to both non-invasive examinations and radiographic examinations. The influence of various modifications to the examination postures applicable in x-ray examination has been described [23]. Unfortunately, that study did not consider postural sway.

Some of the proposed non-invasive methods examine the subject in free standing posture [4,5] while others make use of the original fixation procedures [3,24]. Unfortunately, we were unable to acquire details regarding the influence of measurement positions on both the reduction in postural sway and the changes in spinal curvature. On the other hand, we consider this issue as a very important source of inconsistency between the radiographic and non-invasive measurements of spinal curvature. In addition, we believe this could, at the least, partially prevent widening of the non-invasive method into clinical practice. Knott et al. found reduced variability in measurement using Ortelius 800 (mean difference against x-ray was only 1.5°) when patients were examined using a wide-based stance with hands forward on the wall to fix themselves in more stable position [12].

The need for designing and standardising the posture for spinal shape examination resulted from our experience with the DTP-3 system. At first, we carried out examinations in free standing posture but we found that postural sways are so momentous that they can considerably skew the diagnostic output of the examination. That is why we have begun to search for such measurement posture that could significantly reduce postural sway by means of mechanical fixation of the examined subject. In the previous study, we proposed and assessed the fixation procedures supporting the upper limbs against a wall (posture B) or supporting the chest against a wall (posture C) [15]. We discovered, however, that this type of fixation either does not bring about significant reduction in postural sway (posture B) or significantly influences the curvature of the spine (posture C). In the following study, we proposed the first variant of a fixation frame that supports the front part of the shoulders and we labelled the posture as posture D [17]. For evaluating the maximum reduction in postural sway, we also evaluated posture F – prone lying position. We discovered that posture D offers significant reduction in postural sway in the mediolateral direction, whereas, there was considerable room for improvement in the anterioposterior direction. In the end, the fixation frame was innovated as described in this study and the corresponding posture was labelled as posture G.

The average value of postural sway when lying prone (posture F) is 0.9, 1.3, and 1.0 mm [17]. In fact, posture G is characteristic of similar values. Further on, we should notice that SD values are more or less the same in all directions. That is why we assume that further significant reduction in SD sizes below 1 mm is not possible anymore pursuant to limitations by the respiratory movements of the trunk and by the accuracy of manual setting of the measuring stylus on the designated points of the spinous processes. Random error, expressed as SD 1 mm, is already acceptable for spinal shape examination since the error in the case of palpation of the spinous processes does not have smaller value. Finally, change in the spinal curvature in posture G, compared to free standing posture, is smaller than 5°, which is, in orthopaedic practice, regarded as a tolerable difference between two examinations [25].

## Conclusions

The study shows that the newly developed fixation frame offers significant reduction in postural sway while maintaining the basic spinal curvature values. The residual sway is so minor that a single examination is sufficient for the entire spinal shape examination, representing significant savings on time. In the free standing posture, it was necessary to apply an average of several (e.g. 5) repeated examinations in order to improve the reliability of the examination. On this basis, we believe that the frame can be used in clinical practice to reduce the influence of postural sway on the precision and reproducibility of the examination.

## Abbreviations

3D, Three dimensional; CT, Cervicothoracic junction; DTP-3, Brand name of Czech origin, it can be translated as spinal shape diagnostics; IV, Ideal vertical; MM, Millimetres; MSD, Mean SD; SD, Standard deviations; PSIS, Posterior superior iliac spine; TL, Thoracolumbar junction.

## Competing interests

We are not aware of any potential conflict of interests.

## Authors’ contributions

JK and JG contributed equally to this manuscript; they proposed conception and design of the study and were involved in drafting the manuscript; JK made measurements of spinal shape and statistical analysis; JK, JS and PS contributed significantly to the development of DTP-3 measurement method; PS and JS contributed significantly to development of a new fixation frame. All authors read and approved the final manuscript.

## Authors’ information

All authors are with the Palacky University Olomouc, Czech Republic.

Jakub Krejci, PhD is a researcher at the Department of Natural Sciences in Kinanthropology, Faculty of Physical Culture; Associated Professor Jiri Gallo, MD, PhD is the chief of Department of Orthopaedics, Faculty of Medicine and Dentistry and in parallel with the Teaching Hospital Olomouc, Czech Republic; Petr Stepanik is a technician at the Department of Natural Sciences in Kinanthropology, Faculty of Physical Culture; Associated Professor Jiri Salinger, PhD is a senior researcher at the Department of Natural Sciences in Kinanthropology, Faculty of Physical Culture.
